# Identification of Sporopollenin as the Outer Layer of Cell Wall in Microalga *Chlorella protothecoides*

**DOI:** 10.3389/fmicb.2016.01047

**Published:** 2016-06-30

**Authors:** Xi He, Junbiao Dai, Qingyu Wu

**Affiliations:** MOE Key Laboratory of Bioinformatics, Center for Synthetic and Systems Biology, School of Life Sciences, Tsinghua UniversityBeijing, China

**Keywords:** biodiesel, cell wall, *Chlorophyta*, oil-extraction, sporopollenin

## Abstract

*Chlorella protothecoides* has been put forth as a promising candidate for commercial biodiesel production. However, the cost of biodiesel remains much higher than diesel from fossil fuel sources, partially due to the high costs of oil extraction from algae. Here, we identified the presence of a sporopollenin layer outside the polysaccharide cell wall; this was evaluated using transmission electron microscopy, 2-aminoethanol treatment, acetolysis, and Fourier Transform Infrared Spectroscopy. We also performed bioinformatics analysis of the genes of the *C. protothecoides* genome that are likely involved in sporopollenin synthesis, secretion, and translocation, and evaluated the expression of these genes via real-time PCR. We also found that that removal of this sporopollenin layer greatly improved the efficiency of oil extraction.

## Introduction

Global fossil fuel consumption has been increasing due to both population and economic growth (Lewis and Nocera, 2006), resulting in an energy shortage and a series of environmental problems such as global warming and effluent gas emissions (Chirinos et al., 2006). In response to these challenges, vigorous research programs are underway that to develop alternative biofuels, which are considered necessary for environmental and economic sustainability (Turner, 1999). Unfortunately, to date, biodiesel fuels obtained from plants and animals can't satisfy the existing demand for transport fuels. Microalgae appears to be a particularly promising candidate for use in biodiesel production because of its rapid growth rate, high oil production capacity, and lack of competition for agricultural land (Chisti, 2007). However, the price of algal biofuels remains much higher than that of conventional fossil fuel, due to the high production cost of both biomass and oil extraction (Demirbas and Fatih Demirbas, 2011). *Chlorella protothecoides*, a member of the *Chlorophyta*, can grow autotrophically when light is available. However, when *C. protothecoides* is grown in heterotrophic conditions (limited nitrogen and abundant glucose), it is able to reach very high cell densities (51.2 g L^−1^) and accumulate large amounts of lipids within cells (55.2%; Xu et al., 2006). It is considered to be a good model for research addressing commercial biofuel production. Transition from autotrophic condition to heterotrophic condition can reduce economic costs in biomass production. Nevertheless, oil extraction from *C. protothecoides* continues to be a significant challenge; it is highly energy consuming and because the cells are hard to break without harsh conditions such as the use of a bead beater (Xiao et al., 2015). Presumably, this resistance to cell lysis may result from a special structure and/or from the composition of the cell wall. Such speculations motivated us to analyze the composition of the cell walls of *C. protothecoides*.

Microalgal cell walls are complex and poorly understood. The chlorella intraspecies variation in cell walls can be dramatic and thus it is difficult to predict which of the compounds will be present in any one strain (Gerken et al., 2013). While some chlorella have only a single layer, others have two layers with the microfibrillar layer proximal to the cytoplasmic membrane and a thin mono or trilaminar outer layer (Yamada and Sakaguchi, 1982). Previous studies about the cell walls of different algae have indicated that only a limited number of chlorella species and other green algae are capable of synthesizing recalcitrant cell walls for protection from chemical or bacterial degradation. For example, some taxa can produce sporopollenin (Geisert et al., 1987; Ueno, 2009), a major component in the cell walls of spores; it has been reported in several genera of green algae, including *Chlorella, Scenedesmus, Pediastrum, Chara, Prototheca*, and *Coelastrum* (Burczyk and Czygan, 1983; Komaristaya and Gorbulin, 2006). In addition, Pore et al. found that acid and alkaline hydrolysis of *C. protothecoides* destroyed the cells, but could not destroy the cell wall components (Pore, 1984). Furthermore, the cell wall components were found to be resistant to acetolysis, which lead them to conclude the presence of sporopollenin. However, Lu et al. reported that they could generate protoplasts of *C. protothecoides* successfully using cellulase and snailase, which suggests the absence of sporopollenin (Lu et al., 2012). Therefore, it is arguable whether sporopollenin is present in *C. protothecoides*. Due to the recalcitrant nature of sporopollenin and sporopollenin-like materials, little is known about their definite chemical structure (Brooks and Shaw, 1978; Delwiche et al., 1989). Some studies have described unique structural features (Atkinson et al., 1972), and the conserved biogenesis pathway of sporopollenin has been predicted in some detail (Ríos et al., 2013).

In this paper, we report the discovery that the cell walls of *C. protothecoides* are resistant to the cell wall degradation enzymes. This suggests the presence of a protective layer that presumably prevents enzymes from accessing the wall components. We performed transmission electron microscopy (TEM), 2-aminoethanol treatment, acetolysis, and Fourier Transform Infrared Spectroscopy and provide evidence that this presumed extra layer exists and is composed of sporopollenin. Subsequently, we performed bioinformatics analysis of the sequenced *C. protothecoides* genome to identify genes that are likely involved in sporopollenin biogenesis and analyzed the expression of these genes with real-time PCR methods. In addition, we used a microfluidic device and monitored the propagation of single algal cells in detail. We found that these cells employ the typical *Chlorella* reproduction pattern and that their cell walls contain sporopollenin throughout the entire life cycle. We conclude that this sporopollenin is likely the primary obstacle to efficient oil extraction in this important model algal species.

## Materials and methods

### Strains and culture conditions

*C. protothecoides* sp. 0710 was cultured as described previously (Yan et al., 2011). Briefly, the autotrophic algae was grown at 28°C with continuous illumination at 40 μmol·m^−2^·s^−2^. The heterotrophic algae was grown in basal medium supplemented with 30 g L^−1^ glucose and 2.5 g L^−1^ yeast extract. Cells were incubated at 28°C in flasks with shaking at 220 rpm.

### Enzymatic treatment of cells

Enzymatic treatment was performed as to previous study (Lu et al., 2012), with slight modifications. Log-phase *Chlorella* cells were harvested by centrifugation at 3000 rpm for 5 min, then the cell pellet was suspended in 25 mM Tris buffer (pH 6.0) containing the cell wall degrading enzymes and 0.6 M D-mannitol. Besides using the same combination of cellulase and snailase as described, a list of commercially available enzymes including cellulase (Sigma Cat. No. C1184 and Newprobe R-10), snailase (Newprobe), cellulysin (Calbiochem Cat. No. 219466), hemicellulase (Sigma Cat. No. H2125), pectinase (Sigma Cat. No. P2611), pectolyase (Sigma Cat. No. P3026), lysozyme (Sigma Cat. No. L6876), and zymolase (Zymoreseach Cat. No. E1005), were applied, either individually or in combination, so as to obtain the optimal digestion condition. Each treatment was kept at 30°C for 16 h. The cells were then harvested for further analysis.

### Fluorescence microscopy

Cells, both before and after disruption, were incubated in the enzyme solution containing 2% cellulase and 1% snailase in 0.6 M sorbitol and 0.6 M mannitol at 30°C for 16 h. These cells were placed onto a clean glass slide and one drop of Calcofluor-white stain (Sigma Cat. No. P3543) and one drop of 10% potassium hydroxide were added sequentially to the slide. Following incubated for 1 min, cell wall fluorescence was examined under a confocal microscope (SP5, Leica).

### Cell wall extraction

Cells in the logarithmic growth phase were harvested by centrifugation at 3000 rpm for 5 min. The pellet was washed three times and resuspended in deionized water. Cell wall fragments were isolated as described previously (Hills, 1973; Matias and Beveridge, 2005). The cells were then transferred into a new 2 ml microfuge tube to which 0.3 g acid-washed glass beads (Sigma Cat. No. 18406) were added and processed for 30 times, for 30 s each time, in a Mini-Beadbeater (BioSpec Cat. No. 3110BXEUR) at 5000 rpm. An equal volume of 10% SDS was added, and the samples were boiled for 5 min, followed by centrifugation at 10,000 rpm for 5 min. The supernatant was then removed and the cell wall fragments which form the white fraction on the top of pellets, were isolated and washed three times in deionized water to remove excess SDS before use in subsequent experiments.

### Transmission electron microscopy

Transmission electron microscopic analysis was performed at the microscope facility in School of Life Sciences of Tsinghua University using a Hitachi H7650B transmission electron microscope following previously-described protocols (Burczyk and Hesse, 1981). Related measurements were taken using the metrics tool in illustrator.

### Acetolysis assay

Acetolysis was carried out for 5 min at 95–100°C in a mixture of acetic anhydride and concentrated sulfuric acid at a ratio of 9:1 (V/V). Cells were transferred to glacial acetic acid both before and after acetolysis to protect the acetic anhydride from breakdown.

### Fourier transform infrared spectroscopy

For infrared absorption spectroscopic analysis, the cell wall fragments were first treated with the enzyme mixture as described in the previously-detailed sample preparation protocol to remove the polysaccharide layer of the cell wall. The samples were then processed by washing at 50°C for 5 min followed by treatment with a chloroform-methanol mixture at a ratio of 2:1 (V/V) at 50°C for 5 min. The process was repeated twice. Then the residues were washed sequentially in glacial acetic acid; 0.1 M sodium acetate; 1.0 M sodium hydroxide containing 0.01% tritonX-100 in a boiling water bath for 5 min; 0.1 M sodium acetate; 0.025 M phosphate buffer (pH-7); water; absolute methanol and absolute ether. The samples were then dried overnight in air. Fourier transform infrared spectroscopy (FTIR) analysis was carried out using a Nicolet 6700 FTIR spectrometer coupled with a continuum IR microscope. Operation conditions used a KBr beam splitter and an MCT-A detector (7800–350 cm^−1^). Spectra were obtained in the mid-infrared (4000–400 cm^−1^) region. 32 scans were accumulated for each spectrum, with a spectral resolution of 4 cm^−1^.

### Identification of enzymes involved in sporopollenin biosynthesis

The sporopollenin-related protein sequences of *Arabidopsis* were downloaded from the Arabidopsis information resource^1^ (TAIR). The protein sequences of *C. protothecoides* were then used as a database while the *Arabidopsis* proteins were used as query sequences for local BLASTP searching to identify homologous protein sequences in *C. protothecoides* (Wei et al., 2012). We also used CD-Search, a database^2^ for the functional annotation of proteins (Marchler-Bauer et al., 2005), to identify conserved domains among the protein sequences of the two organisms.

The whole genome sequence has been deposited at DDBJ/EMBL/GenBank under the accession identifier APJO00000000. The sequences for sporopollenin synthesis-related genes in *C. protothecoides* have been deposited to the GenBank nucleotide database under the following accession identifiers: KF517419 (Cpr000314.3), KF517420 (Cpr001396.1), KF517421 (Cpr000450.1), KF517422 (Cpr002802.1), KF517423 (Cpr000351.1), KF517424 (Cpr004505.1), KF517425 (Cpr001179.1), KF517426 (Cpr002918.1), KF517427 (Cpr001636.6), KF517428 (Cpr004904.1), KF517429 (Cpr001668.1), KF517430 (Cpr004207.2), and KF517431 (Cpr002170.1).

### Real-time quantitative PCR

Freshly harvested *C. protothecoides* cells were quickly frozen and grounded until the liquid nitrogen evaporated. Total RNA was then isolated using Trizol Reagent (Invitrogen, Cat. No. 15596-026) and genomic DNA was removed by treatment with RNase-free DNaseI (Takara, Cat. No. 2270A). First-strand cDNA was prepared from 4 μg of total RNA using Reverse Transcriptase XL (Takara, Cat. No. 2621) according to the product protocol. PCR amplification was performed using primers designed with Primer Premier 5.0. All primers are listed in Table S2. Actin was used as the internal control. Fluorescence-based real-time PCR reactions were performed in an optical 96-well plate with a Roche LightCycler®480 II Detection System using SYBR® Green (Invitrogen, Cat. No. 4385618). The 2^−ΔΔCt^ method was used to calculate the changes in relative gene expression measured from the real time quantitative PCR analyses.

### Single cell analysis of asexual reproduction in *C. Protothecoides* using a microfluidic device

We used a microfluidic system capable of retaining algae cells in microfluidic chambers; this system has been used successfully in yeast for related studies (Tian et al., 2013). This system enables the observation of the asexual reproduction process in single cells throughout their lifespan. The microfluidic device was kindly designed and provided by Chunxiong Luo of Peking University. Images were taken with a Nikon TE2000 time-lapse microscope; bright field images were taken once every 20 min.

### Measurement of oil content

Lipid composition and accumulation were analyzed via GC-MS using a slightly modified version of our previously-published protocols (Lu et al., 2010). Wet algal cells treated with or without 2-aminoethanol were suspended in a mixture of 0.5 ml methanol acidified with 3% sulfuric acid and 0.5 ml chloroform containing 2.5 g L^−1^ capric acid (used as an internal standard to correct transesterification and injection volume errors). The mixture was extracted for different durations of time and then centrifuged at 6000 rpm for 5 min. The supernatant with 3 ml mixture (1.5 ml methanol acidified with 3% sulfuric acid and 1.5 ml chloroform containing 2.5 g L^−1^ capric acid) was then heated in a sealed tube at 70°C for 2 h. After cooling, 1 ml of distilled water was added and the sample was vortexed for 20 s. After separation, 1 μl of the lower phase was injected into a gas chromatograph (HP 689-, USA) using a 30 m, 0.32 mm diameter column. Nitrogen was used as the carrier gas at a flow rate of 1 ml min^−1^. Measurements started at 80°C for 1.5 min. The temperature was increased to 140°C at a rate of 30°C min^−1^. The next step of the method increased the temperature at a rate of 20°C min^−1^ to 300°C and held this temperature for 1 min before the analysis was terminated. The Retention times for the analytes were as follows: 4.4 min for capric acid methyl ester, 7.4 min for hexadecanoic acid, and 8.5 min for octadecanoic acid methyl ester.

## Results

### The cell wall of *C. Protothecoides* includes one layer resistant to cellulose degrading enzymes

**Figure 1:**
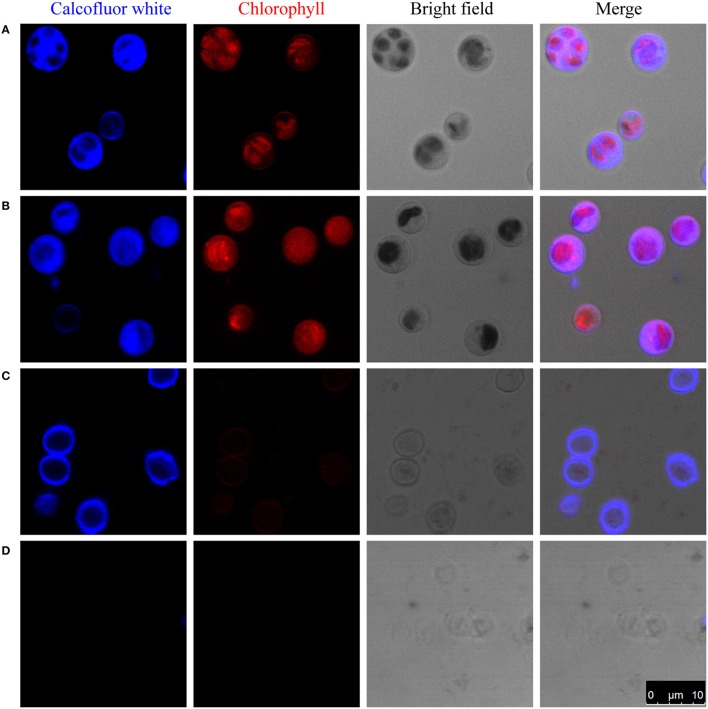
**Enzymatic digestion and Calcofluor-white staining of the cell wall of *C. protothecoides***. The blue fluorescence indicates the presence of a cell wall that contains cellulose or chitin. The red fluorescence is chloroplast autofluorescence. **(A)** Intact cells; **(B)** Intact cells after enzymatic treatment; **(C)** Disrupted cells; **(D)** Disrupted cells after enzyme treatment. Arrows show cell wall fragments after enzyme treatment.

To study the composition of cell walls, we first stained algal cells with a commonly used cell wall dye, Calcofluor-white, which binds to cellulose and chitin (Hughes and McCully, 2009). As shown in Figure 1A, whole cells stained with Calcofluor-white showed an intense, light blue fluorescence when irradiated with UV, suggesting that the cell walls of *C. protothecoides* contain cellulose or chitin, similar to other algae (Popper and Fry, 2003). The enzymatic digestion of algal cell walls has been used for a long time in algal research for the preparation of protoplasts (Aach et al., 1978). Lu et al. described a method for generating protoplasts from *C. protothecoides* using an a enzyme combination of 2% cellulase and 1% snailase (Lu et al., 2012). We therefore used the same combination of enzymes to treat the *Chlorella* cells in this study. However, this treatment failed to remove the cell wall (Figure 1B). Protoplasts of *Chlorella saccharophila, Chlorella ellipsoidea* (Braun and Aach, 1975; Yamada and Sakaguchi, 1982), and *Chlorella vulgaris* (Yang et al., 2015) were obtained using an enzyme mix containing cellulase, hemicellulose, and pectinase. We acquired a number of commercially available cell wall degrading enzymes, including cellulase, cellulysin, snailase, hemicellulase, pectinase, pectolyase, lysozyme, and zymolase, and applied these, both individually and in various combinations. None of these enzymes could generate protoplasts from *C. protothecoides* (Table S1).

One of the possibilities for the inability to get protoplasts is that these enzymes are somehow prevented from accessing the cellulose/chitin layer. To test this hypothesis, we partially disrupted the cells in mild to moderately harsh conditions and then stain the sample cells. Pre-disruption of cells with a bead beater in mild conditions retained a round globular structure and showed light blue fluorescence when stained with Calcofluor-white, indicating the presence of a cellulose/chitin layer in the cell wall around these cells (Figure 1C). Interestingly, following treatment with an enzyme combination of 2% cellulase and 1% snailase, most cells failed exhibit fluorescence upon Calcofluor-white staining, suggesting the loss of cellulose/chitin layer around these cells (Figure 1D). These results indicate that cellulose/chitin is one of the major components of the *C. protothecoides* cell wall and is protected from enzymatic degradation by a hitherto unknown mechanism.

### Identification of sporopollenin in the cell wall of *C. Protothecoides*

**Figure 2:**
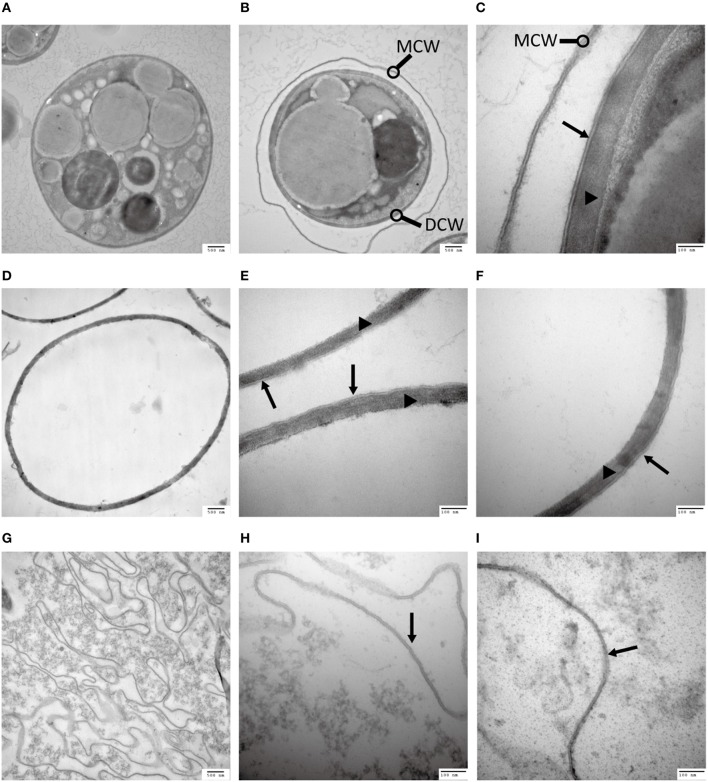
**Analysis of the *C. protothecoides* cell wall under TEM. (A)** Intact heterotrophic *C. protothecoides* (× 30K). **(B)** The same with **(A). (C)** Was similar with **(A,B)** but at higher magnitude (× 120K). **(D)** Cell wall before enzymatic treatment (× 30K). **(E)** Was similar with **(D)** but at higher magnitude (× 120K). **(F)** The same with **(E). (G)** Cell wall after enzymatic treatment. (× 30K). **(H)** Was similar with **(G)** but at higher magnitude (× 120K). **(I)** The same with **(H)**. DCW, daughter cell wall; MCW, mother cell wall; Triangles show the inner layer of the cell wall; Arrows show the outer layer of the cell wall.

In a quest to understand what prevents the cell wall being subjected to enzymatic degradation, we examined the ultra-structure of the *C. protothecoides* cell wall using TEM. Isolated cell walls were subjected to TEM analysis, at different magnitudes, both for cells cultured heterotrophically (Figure 2) and for cell cultured autotrophically (Figure S1). As shown in Figures 2A–F, the cell wall of heterotrophic *C. protothecoides* consists of two morphologically distinct components that are referred to henceforth as the “inner” and “outer” parts of the wall. The inner part is similar to the cell wall of many plants and presumably consists of polysaccharides and proteins. The inner wall of heterotrophic algae (Figures 2C–F) is about 80–100 nm in thickness; wider than the inner part of the walls of autotrophic algae cells, which is about 50–60 nm (Figures S1C–F). A mixture of 2% cellulase and 1% snailase was about to completely digest the inner wall of both heterotrophic (Figures 2G–I) and autotrophic algae (Figures S1G–I). This observation suggested that the inner wall is probably composed of cellulose and protein, similar to the cell walls of other plants or algae (Lora et al., 2014). It is known that the polysaccharide components in the cell wall can support the structure and keep cells in a relatively constant circular state, so it was not surprising that the loss of polysaccharides following enzymatic treatment resulted in curved cell wall fragments (Figure 2G, Figure S1G).

**Figure 3:**
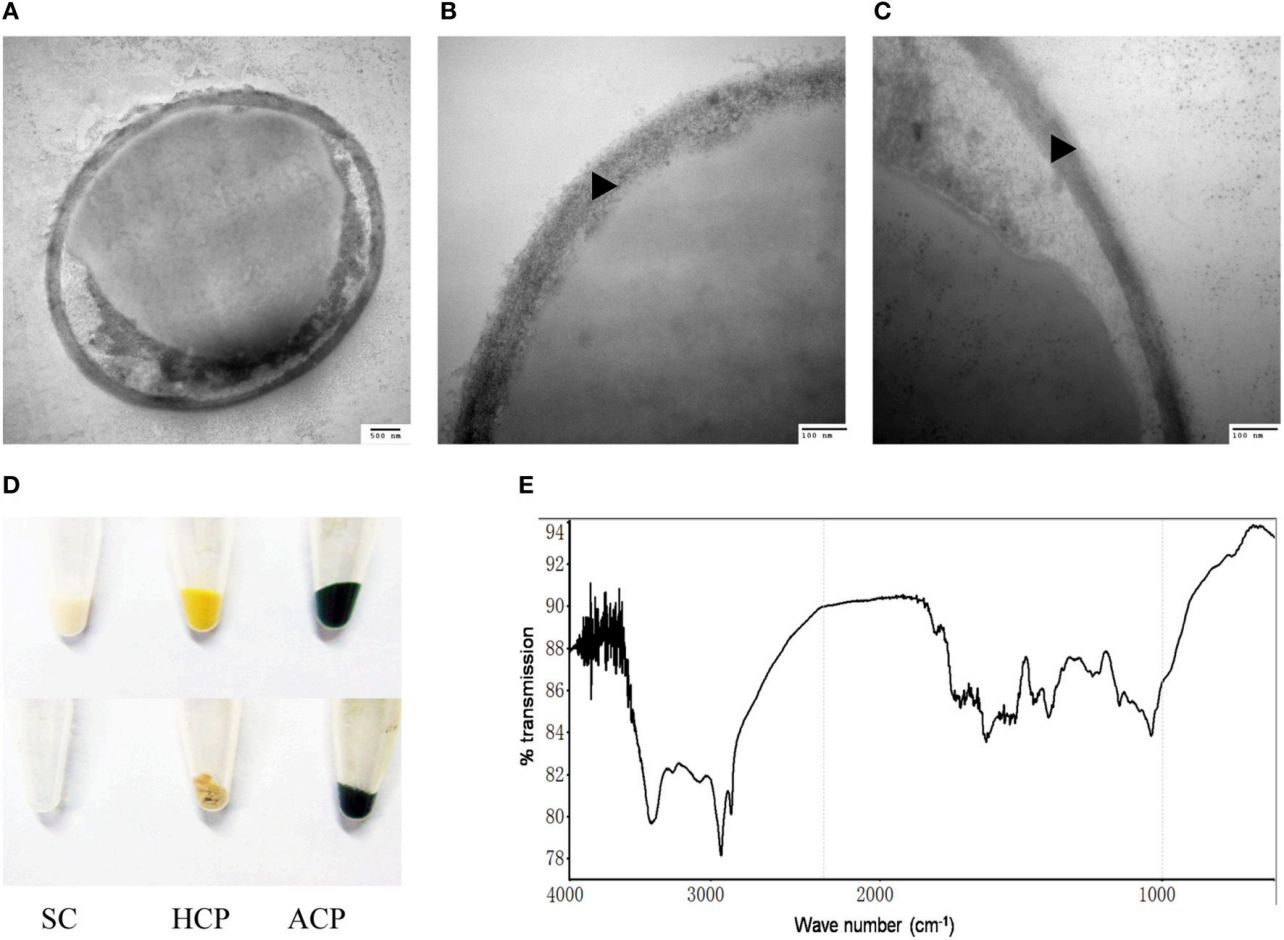
**Identification of sporopollenin in the cell wall of *C. protothecoides*. (A)** Following 2-aminoethanol treatment were examined using TEM (× 30K). **(B)** The same with **(A). (C)** Similar with **(A,B)** but at higher magnitude (× 120K). **(D)** Acetolysis of *C. protothecoides* cells. Samples from left to right represent yeast, heterotrophic algae, and autotrophic algae. Samples before Acetolysis treatment were on the top and after treatment were at the bottom. **(E)** FTIR of the cell wall of *C. protothecoides*. SC, Saccharomyces cerevisiae; HCP, Heterotrophic *C. protothecoides*; ACP, Autotrophic *C. protothecoides*. Triangles show the inner layer of the cell wall.

The outer layer of both autotrophic and heterotrophic algae displayed a trilaminar composition and was about 10 nm in thickness (Figure 2C). It was sandwich-like in structure and included an electron-lucent center layer and two electron-dense zones on either side of this layer. Interestingly, the outer layer was resistant to enzymatic digestion, indicating that it was probably not made of cellulose or other polysaccharides. Previous studies have suggested that the outer layer of pollen from many plants is resistant to enzymatic digestion and is composed of sporopollenin, a material that can be dissolved in oxidizing solutions, fused potassium hydroxide, or 2-aminoethanol (Southworth, 1974; Domínguez et al., 1999). Therefore, we treated the isolated cell walls with 2-aminoethanol and found that the outer layer became smeared although the inner layer did not undergo significant changes (Figures 3A–C). This experiment that there is an outer layer of the *C. protothecoides* cell wall that is possibly composed of sporopollenin.

To evaluate whether or not the newly-identified layer is indeed made of sporopollenin, two additional assays were performed. First, the algal cells were subjected to acetolysis, a process to which sporopollenin is known to be resistant (Heslop-Harrison, 1969). As a control, yeast cells were treated under the same conditions. As shown in Figure 3D, the yeast cells were completely dissolved following acetolysis, resulting in a transparent solution. With the *C. protothecoides* samples, however, a large amount of insoluble material remained after treatment. This result is similar findings of Good and Chapman, who identified sporopollenin-containing plants (Good and Chapman, 1978). We next analyzed the composition of the outer layer directly using infrared spectroscopy. We observed two large peaks around the 3000 cm^−1^ wavelength, and noted a slight variation from 1400 to 1500 cm^−1^ (Figure 3E, Figure S2). This spectral data matches the previously-reported spectra of pollen as well as the outer layer of the cell walls of other sporopollenin-containing algae (Watson et al., 2007). Taken together, these results strongly support the assertion that sporopollenin is present in the outer layer of the cell walls of *C. protothecoides*.

### Identification of genes likely involved in sporopollenin biosynthesis in the genome of *C. Protothecoides*

**Figure 4:**
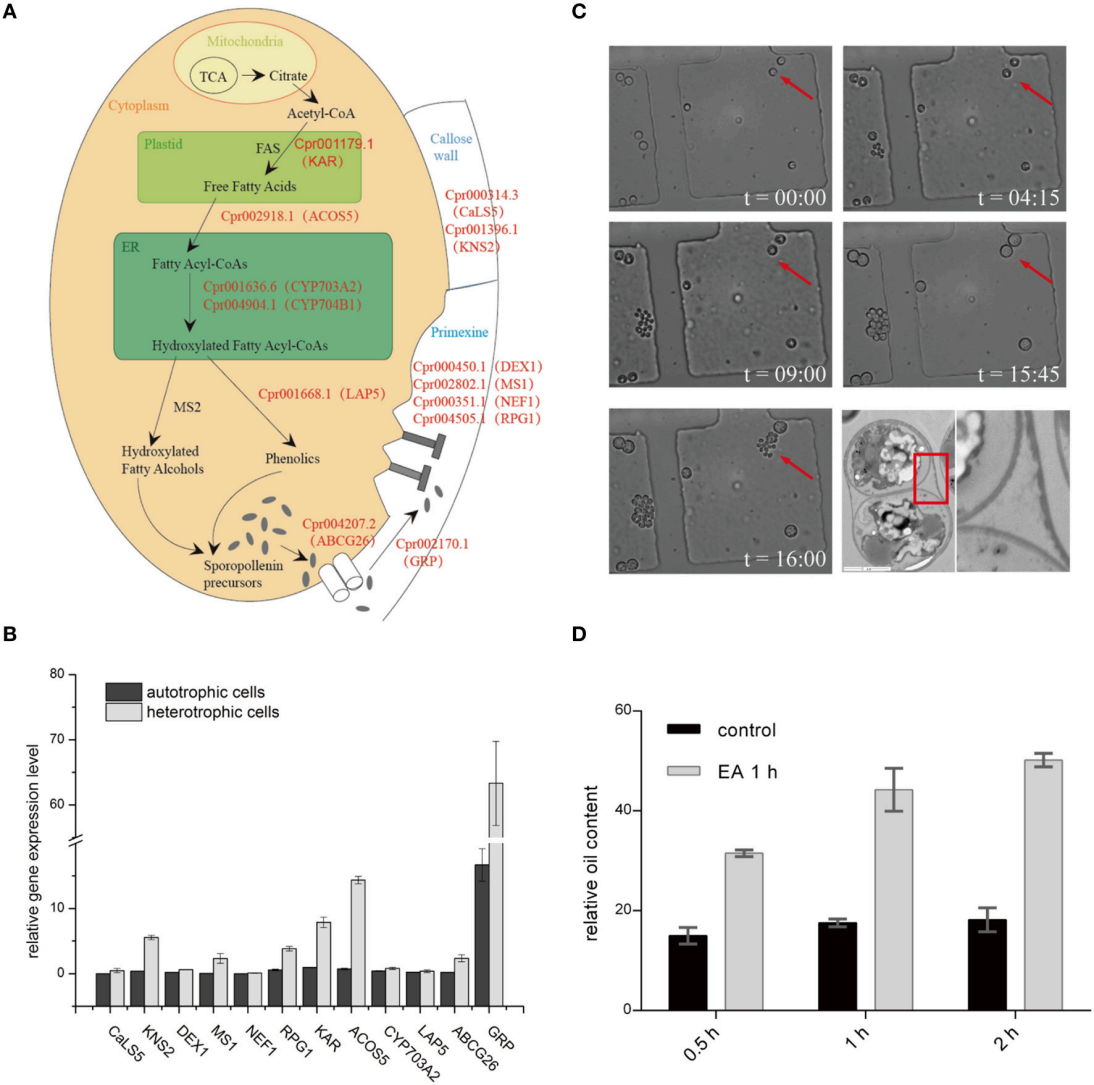
**Sporopollenin synthesis pathway in *C. protothecoides*. (A)** A schematic model of sporopollenin biosynthesis in *C. protothecoides*. **(B)** Expression of genes likely required for the synthesis, secretion, and translocation of sporopollenin in autotrophic and heterotrophic cells. Selected genes from Table 1 were tested in this assay. Error bars represents the standard error from two independent experiments. **(C)** The continuous tracking of algal cells in a microfluidic device monitored patterns and dynamic changes in cell walls during asexual reproduction in *C. protothecoides*. The time point of cell injection into the device was set as the starting point (*t* = 00:00). Images were taken every 15 min; only selected time points are shown. A representative TEM of a dividing cell is also shown. **(D)** Effects of 2-aminoethanol treatment on oil extraction efficiency. Wet algal cells, with or without 2-aminoethanol treatment, were suspended in the extraction mixture for 0.5, 1, or 2 h.

Genomic analysis of sporopollenin formation in higher plants has indicated that sporopollenin shares a common synthesis pathway, and a schematic model of sporopollenin formation has been proposed (Ma, 2005; Blackmore et al., 2007). We recently published a draft genome of *C. protothecoides* (Gao et al., 2014). This genome enabled us to identify genes in this alga that are homologous to enzymes known or thought to function in key roles in the sporopollenin synthesis pathway. Using *Arabidopsis* homologs as reference, we found that almost all of the genes known to be associated with sporopollenin formation could be identified in the *C. Protothecoides* genome (Table 1); further analysis resulted in the identification of conserved domains among these enzymes. The key genes for sporopollenin formation are visually summarized as three steps in *C. protothecoides*, as shown in Figure 4A. First, the formation of a callose wall that separates individual daughter cells; this is likely catalyzed by Cpr000314.3 and Cpr001396.1, which are the homologs, respectively, of CaLS5 and KNS2 in *Arabidopsis*. Pollen wall development is subsequently initiated through primexine formation around distinct daughter cells. Previous studies in plants have shown that that primexine functions as a sporopollenin receptor; four enzymes, DEX1, MS1, NEF1, and RPG1 are required for sporopollenin deposition and polymerization (Gabarayeva et al., 2009). Similarly, Cpr000450.1, Cpr002802.1, Cpr000351.1, and Cpr004505.1, which correspond, respectively, to the genes that encode these enzymes, were identified in the genome of *C. protothecoides*. The third step is the synthesis, secretion, and translocation of sporopollenin precursors, a process that is dependent on the synthesis of free fatty acids and their derivatives (Meuter-Gerhards et al., 1999, Figure 4A). Multiple enzymes are known to function in these metabolic pathways, and most of these were found in the *C. protothecoides* genome (Table 1). Genetic studies in *Arabidopsis* and other organisms have shown that mutations in the aforementioned genes, including, for example *CaLS5* and *KNS2*, lead to defects in primexine formation (Ariizumi et al., 2004; Dobritsa et al., 2010; Li et al., 2010). The identification of similar genes in the genome of *C. protothecoides* establishes a putative molecular basis for the existence of sporopollenin in this alga.

**Table 1:** **Identification and analysis of likely sporopollenin synthesis-related genes in *C. protothecoides* using Arabidopsis genes as a reference**.

**Function In Arabidopsis**	**Arabidopsis gene**	**TAIR Reference sequence**	**Proposed Gene class**	**Sequence of best match**	**No. of Proposed Homologs**	**Bits of best match**	**E–scores**	**Protein conserved domains**
CWS	CaLS5	AT2G13680.1	glucan synthase	Cpr000314.3	2	399	e^−111^	glucan_synthase,FKS1
CWS	KNS2	AT5G11110.1	Sucrose phosphate synthase	Cpr001396.1	2	157	e^157^	sucrose phosphate synthase
PS	DEX1	AT3G09090.1	membrane protein	Cpr000450.1	1	277	5e^−075^	VCBS
PS	MS1	AT5G22260.1	Transcription factor	Cpr002802.1	2	95	3e^−02^0	PHD-finger
PS	NEF1	AT5G13390.1	membrane protein	Cpr000351.1	1	69	4e^−012^	/
PS	RPG1	AT5G40260.1	membrane protein	Cpr004505.1	2	54	2e^−008^	MtN3_slv superfamily
SST	KAR	AT1G24470.1	glucose/ribitol dehydrogenase	Cpr001179.1	10	213	e^−065^	17beta-HSD1_SDR_c, NADB_Rossmann superfamily
SST	ACOS5	AT1G62940.1	lipid metabolism	Cpr002918.1	11	135	e^−168^	FACL,AFD_Class_I superfamily
SST	CYP703A2	AT1G01280.1	cytochrome P450	Cpr001636.6	7	107	5^*e*−024^	p450 superfamily
SST	CYP704B1	AT1G69500.1	cytochrome P450	Cpr001636.6	10	116	8^*e*−027^	p450 superfamily,CypX
SST	LAP5	AT4G34850.1	acyl groups transferase	Cpr001668.1	2	52	e^−007^	CHS_like,cond_enzymes superfamily, PLNO3169
SST	ABCG26	AT3G13220.1	ATP-binding transporter	Cpr004207.2	35	344	2^*e*−095^	ABCG_EPDR,ABC2_membrane superfamily
SST	GRP	AT4G38680.1	glycine rich protein	Cpr002170.1	1	80	3^*e*−016^	CSP_CDS,S1_like superfamily

*CWS, Callase wall Synthesis pathway; PS, Primexine Synthesis; SST, Sporopollenin synthesis secretion and translocation*.

To further verify the existence of above identified genes related to sporopollenin biosynthesis, primers were designed according to the sequences of the conserved domains found in *C. protothecoides* genome; PCR products of the expected sizes were amplified from *C. protothecoides* genomic DNA (Figure S3). Additionally, real time PCR was used to compare the expression of sporopollenin-synthesis genes in autotrophic and heterotrophic cells. As shown in Figure 4B, transcripts of all of these genes could be detected under both conditions. Of note, as compared to the cells growing in the autotrophic condition, the heterotrophic cells tended to have higher expression of these genes. It is possible that cells growing in the heterotrophic condition undergo relatively more rapid replication and that relatively higher abundances of enzymes are therefore required to satisfy growth and division demands.

### The sporopollenin layer exists throughout the entire asexual life cycle and forms an obstacle for oil extraction

The continuous tracking of algal cells in a microfluidic device makes it possible to study the cell division dynamics of single cell with high temporal resolution. We monitored a time course of *C. protothecoides* cell division and observed that protoplasts divide into two, four, eight, and so on, within the mother cell wall (Figure 4C). When the mother cell wall ruptures, the daughter cells emerge from the autosporangium; this is a common mode of propagation for unicellular green algae. We present the entire process of the cell cycle arranged into a movie (Movie S1). Algae growing in both autotrophic and heterotrophic media showed the same division pattern (Figure S4). This propagation mechanism immediately suggests that an intact cell wall will be formed for each daughter cell within a mother cell; this was confirmed by transmission electron microscopy analysis (Figure 4C). This also suggests that it is not possible to obtain cells without this sporopollenin layer via targeted collection of mother cells. Therefore, an additional method to remove the sporopollenin layer might be required in order to improve the efficiency for both transformation and oil extraction.

To test this hypothesis, *C. protothecoides* cells were pre-treated with 2-aminoethanol, which was able to dissolve sporopollenin in the aforementioned experiments in our study and in previously-reported experiments (Southworth, 1974; Domínguez et al., 1999, Figure 3A). As expected, we found that the oil yield was significantly improved after 2-aminoethanol treatment as compared the no treatment control cells (Figure 4D). As we reported before (Xiao et al., 2015), oil extraction from *C. protothecoides* is one of the most energy-consuming steps in large scale biofuel production procedures. Our results suggest that a potential solution to help reduce the cost of this step would be the pre-treatment of cells with 2-aminoethanol.

## Discussion

Although, *C. protothecoides* is regarding a good candidate species for biofuel production (Heredia-Arroyo et al., 2010), its cells are notorious their hard-to-disrupt cell wall. This results in an increased cost for oil extraction from these algal cells. According to previous oil production studies, a pre-treatment process is always required to enhance the efficiency of lipid extraction (Halim et al., 2012). Currently, however, no mature pre-treatment methods can be directly applied to *C. protothecoides* to extract oil at an industrial scale. Actually, when typical methods are used, the recovery rate of lipids from *C*. *protothecoides* is not as high as that of other organisms (Shen et al., 2009). It has been a long-standing mystery as to why this particular alga is so resistant to mechanical stress. Several attempts to genetically transform *C. protothecoides* have been unsuccessful. Common methods such as the use of polyethylene glycerol following spheroplast generation, which are used to transform protoplasts of other yeast and fungal cells (Kindle, 1990), do not work for *C. protothecoides*. And direct enzymatic digestion using cellulase, snailase or other cell wall-degrading enzymes cannot remove the *C. protothecoides* cell wall (Figures 1B, 2B,D). The identification of sporopollenin as a major component of the cell wall provides a reasonable explanation to these questions.

Knowing the composition of cell wall should greatly advance the use of *C. protothecoides* as model system for biofuel production. For example, additional pre-treatment steps can be adopted to remove sporopollenin prior to oil extraction, likely leading to increased oil yields. In addition, the identification of the sporopollenin layer promises to facilitate the development of new methods for the genetic transformation of *C. protothecoides*. A previous study suggested that microprojectile bombardment could be used to deliver DNA into *tobacco* pollen to study the expression of pollen-expressed genes (Twell et al., 1989). Given the superficially similar cell wall structure between *C. protothecoides* cells and pollen, it is conceivable that a similar method might also work to transform this alga. Furthermore, screens can be carried out to find mutants in which the synthesis of sporopollenin is blocked. Alternatively, some specific chemicals may be used to inhibit sporopollenin synthesis. These sporopollenin deficient strains may become susceptible and easy to accept foreign DNA.

Recently, an elegant study that systematically treated various *Chlorella* strains with a large number of commercially available enzymes found that *Chlorella* is most sensitive to chitinase and lysozymes (Gerken et al., 2013). We performed a similar series of enzymatic treatments with *C. protothecoides*. Unfortunately, none of the enzymes we tested were capable of removing the cell wall of *C. protothecoides*. This frustrating result actually lead us to carefully examine its cell wall with electron microscopy, leading to our discovery of the sporopollenin layer (Figure 2C).

Sporopollenin is considered to be one of the toughest materials in nature and is known to be resistant to many kinds of chemical and biological attacks. It has been found in *Characium terrestre, Enallax coelastroides*, and *Scotiella chlorelloidea* as a high efficient barrier against physical or chemical damage and has been extremely important in the evolution of terrestrial plants (Xiong et al., 1997). However, only a few green algae species have been reported to contain sporopollenin in their cell walls (Northcote et al., 1958; Krienitz et al., 1999). The function of sporopollenin in microalgae has been proposed to be associated with resistance to environmental stress such as UV irradiation, desiccation, microbiological attack, and so on (Strohl et al., 1977). Similarly, the presence of sporopollenin in the cell wall may help *C. protothecoides* to withstand various environmental stresses.

## Author contributions

QW, JD conceived research; JD, XH designed experiments; XH performed experiments and analyzed the data; all authors wrote, commented on, and approved the contents of the manuscript.

### Conflict of interest statement

The authors declare that the research was conducted in the absence of any commercial or financial relationships that could be construed as a potential conflict of interest.

## Footnotes

^1^http://www.arabidopsis.org/

^2^http://www.ncbi.nlm.nih.gov/entrez/query.fcgi?db=cdd
