# Supplementary material for: Identification of Sporopollenin as the Outer Layer of Cell Wall in Microalga Chlorella protothecoides
Source: Front Microbiol. 2016 Jun 30;7:1047. doi: 10.3389/fmicb.2016.01047 (PMC4928020; doi:10.3389/fmicb.2016.01047)
Supplement: Supplementary file 1 [file Table1.DOCX]

**Table S1 Different enzymes were used for induction of protoplast**

| **Enzyme solution** | **Work condition** |
| --- | --- |
| 2% cellulose  1% snailase  2% cellulysin  2% hemicellulose  50 units pectinase  2% pectolyase  2% lysozyme  2% zymolase  2% cellulose + 1% snailase  2% cellulose + 1% snailase + 2% cellulysin  2% cellulose + 1% snailase + 2% hemicellulose  2% cellulose + 1% snailase + 50 units pectinase  2% cellulose + 1% snailase + 2% pectolyase  2% cellulose + 1% snailase + 2% lysozyme  2% cellulose + 1% snailase + 0.5 mg/ml zymolase | 30^o^C 16 h |
