# Supplementary material for: Identification of Sporopollenin as the Outer Layer of Cell Wall in Microalga Chlorella protothecoides
Source: Front Microbiol. 2016 Jun 30;7:1047. doi: 10.3389/fmicb.2016.01047 (PMC4928020; doi:10.3389/fmicb.2016.01047)
Supplement: Supplementary file 2 [file Table2.DOCX]

**Table S2** **Primer sequences for real-time RT-PCR**

| **Name** | **Sequence (5’-3’)** | **PCR product (bp)** |
| --- | --- | --- |
| Actin | S: GGCATGGGCCAGAAGGA | 299 |
|  | As: GAGGCGTACAGGGACAGCA |  |
| CaLS5 | S: GCAAGCCCGAGAACCAGA | 190 |
|  | As: CGGAAGCCGACCAGTACGAC |  |
| KNS2 | S: CTGCACTTCCGCCGCTCCTA | 123 |
|  | As: GGCCTCGAACTCTTTGACCAC |  |
| DEX1 | S: GTATGTGCTGAATGCGGTGAC | 202 |
|  | As: GCTGATGGCAGACTCAAGGTG |  |
| MS1 | S: CCACCGCCATTTCCACAGA | 115 |
|  | As: CGCCCAAAGTCAAAGCCAAA |  |
| NEF1 | S: CCTGAGGCAGAAGGAGATGGC | 138 |
|  | As: GCAGTGATGAGCAAGACGAGGG |  |
| RPG1 | S: TGTCCTGCTCATGGCCTACT | 239 |
|  | As: CTGCCTGCCCGTTCTTG |  |
| KAR | S: GCCTCATCGTCAACGTGGGA | 135 |
|  | As: CGCCGAACTCGCGGTATT |  |
| ACOS5 | S: CTGAATGAGGAGGGCAAGCG | 120 |
|  | As: GGGCAGTTCGTCGGTGATG |  |
| CYP703A2 | S: CTGTCCACCAACGCATCAAA | 260 |
|  | As: GCCAGGCGGGAGAAGAA |  |
| LAP5 | S: TGCGTGTACCAGCAGGAGG | 163 |
|  | As: GCGCCATCAGGTTGAGGAA |  |
| ABCG26 | S: CGGATGATGCTGAAGGAG | 270 |
|  | As: GATGGTGGCGTAGGTCTG |  |
| GRP | S: GGTGGTGAGGACCTGTTTGTT | 110 |
|  | As: CGGCCATCGTCGGAGTT |  |
