# Supplementary figures and images for: Identification of Sporopollenin as the Outer Layer of Cell Wall in Microalga Chlorella protothecoides
Source: Front Microbiol. 2016 Jun 30;7:1047. doi: 10.3389/fmicb.2016.01047 (PMC4928020; doi:10.3389/fmicb.2016.01047)

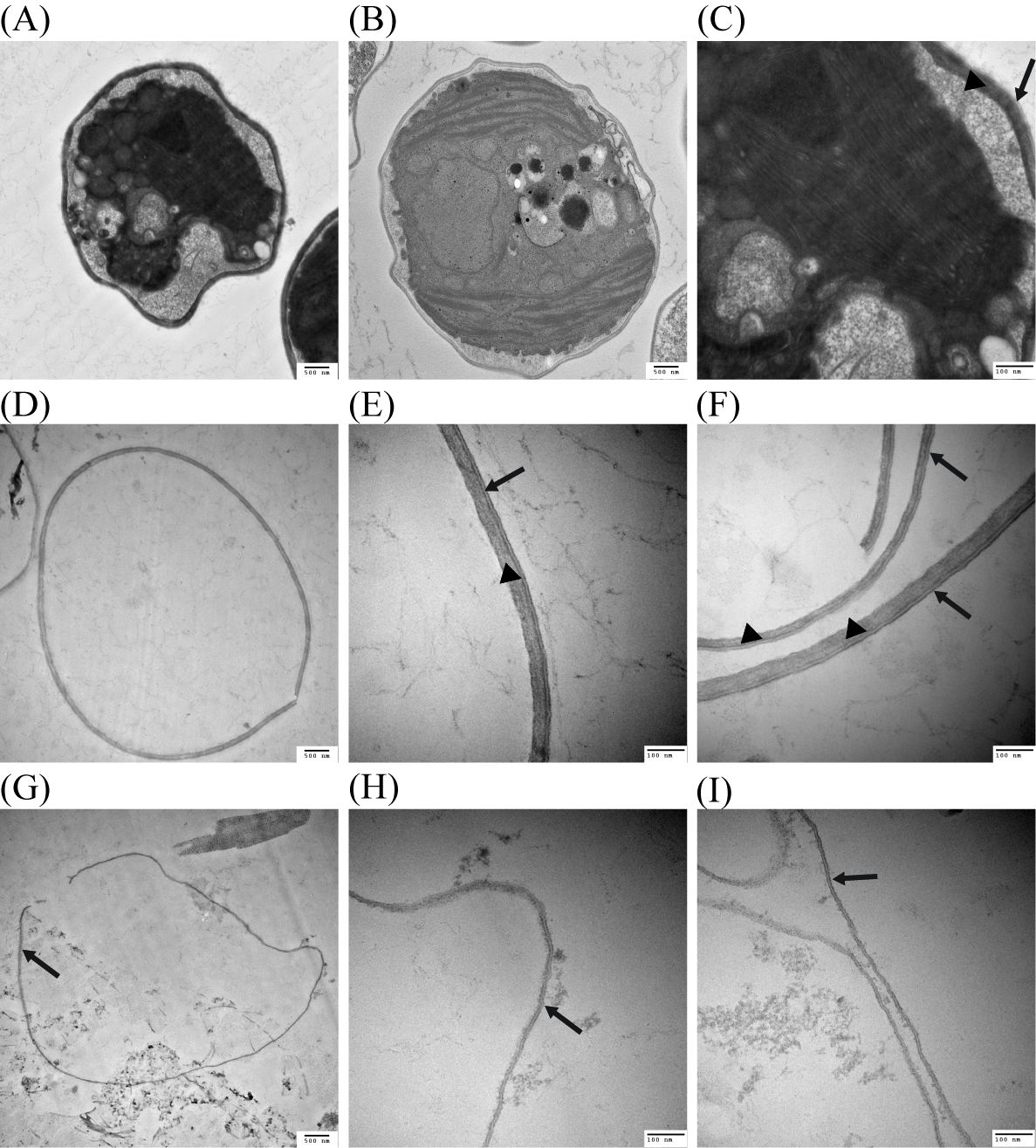

Supplement: Supplementary file 4 [file Image1.TIF]

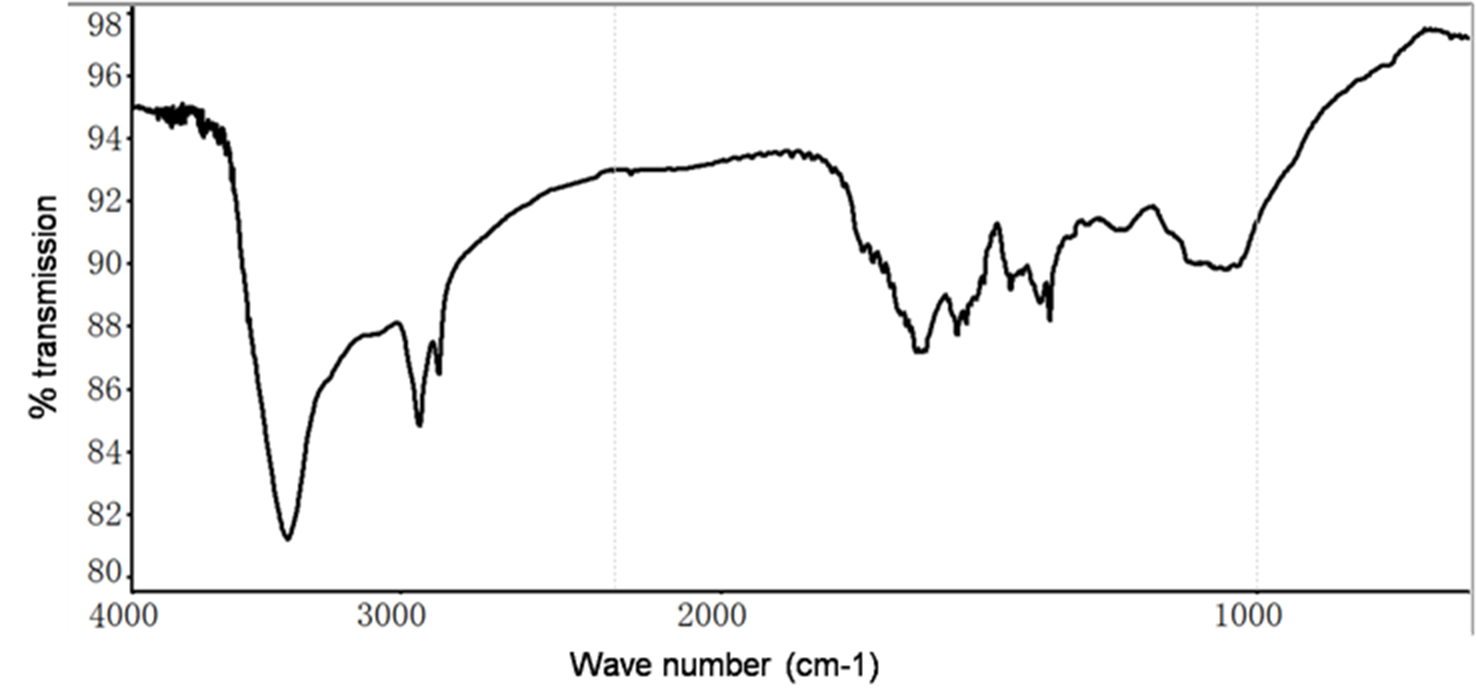

Supplement: Supplementary file 5 [file Image2.TIF]

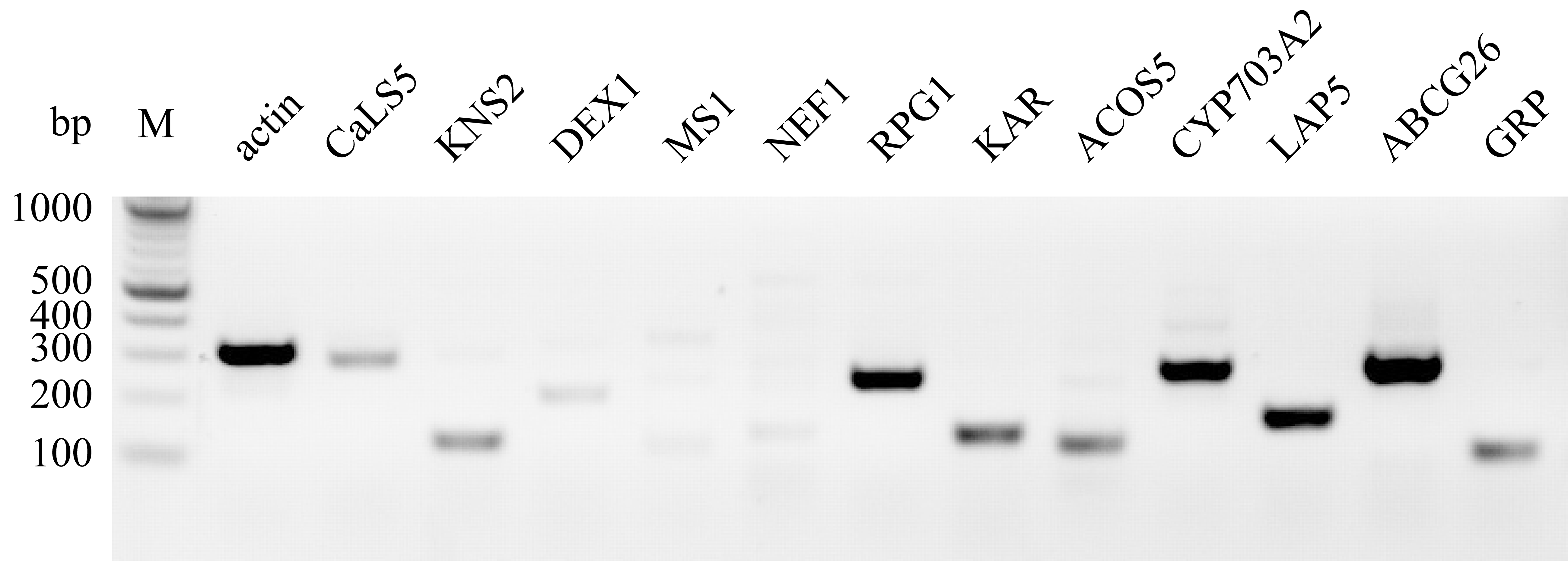

Supplement: Supplementary file 6 [file Image3.TIF]

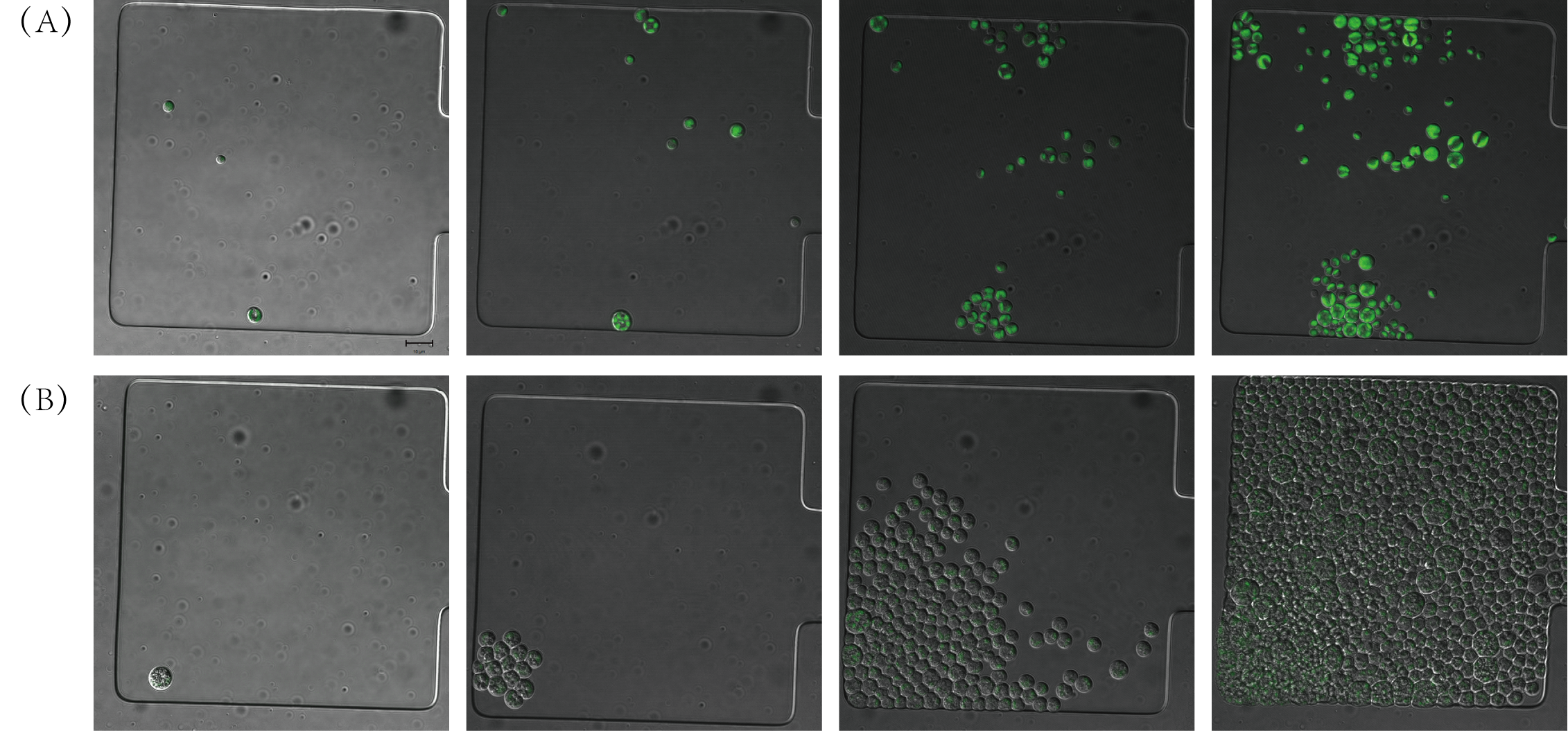

Supplement: Supplementary file 7 [file Image4.TIF]
